# Mitochondrial genome amplification of avian haemosporidian parasites from single-infected wildlife samples using a novel nested PCR approach

**DOI:** 10.1007/s00436-023-07986-1

**Published:** 2023-10-03

**Authors:** Sandrine Musa

**Affiliations:** University of Hohenheim, Emil-Wolff-Str. 34, 70599 Stuttgart, Germany

**Keywords:** *Plasmodium*, *Haemoproteus*, *Leucocytozoon*, *cox3*, *cox1*, *cytb*

## Abstract

Haemosporidian parasites that infect birds (Apicomplexa: Haemosporida) are blood parasites that require an invertebrate host (vector) and a vertebrate host for their lifecycle and cause malaria-like diseases. This group of parasites has provided valuable insights into host specificity, virulence, and parasite dispersal. Additionally, they have played a significant role in reshaping our understanding of the evolutionary history of apicomplexans. In order to accurately identify species and to address phylogenetic questions such as the timing of the haemosporidian radiation, the use of a sufficiently large genetic data set is crucial. However, acquiring this genetic data poses significant challenges. In this research, a sensitive nested PCR assay was developed. This assay allows for the easy amplification of complete mitochondrial genomes of haemosporidian parasites in birds, even during the chronic stage of infection. The effectiveness of this new nested PCR assay was evaluated using blood and tissue samples of birds with verified single parasite infections from previous studies. The approach involves amplifying four overlapping fragments of the mitochondrial genome and requires DNA extracts from single-infected samples. This method successfully amplified the complete mitochondrial genomes of 24 distinct haemosporidian parasite lineages found in various bird species. This data is invaluable for conducting phylogenetic analyses and accurately defining species. Furthermore, this study proposes the existence of at least 15 new haemosporidian parasite species based on the genetic information obtained. Data regarding pGRW04, previously categorized as *Plasmodium relictum* like pSGS1 and pGRW11, indicates that the pGRW04 lineage is actually a separate, hidden *Plasmodium* species.

## Introduction

Avian haemosporidian parasites (Apicomplexa: Haemosporida) represent obligate heteroxenous blood parasites, reliant on dipteran vectors for transmission, and responsible for inducing malaria-like diseases in avian hosts. This taxonomic group comprises three genera—*Plasmodium*, *Haemoproteus*, and *Leucocytozoon*—with a global distribution, often linked to diminished host fitness and mortality among susceptible bird species (Valkiunas 2005). In addition to shedding light on host specificity, virulence, and parasite dispersion, this parasite consortium has been instrumental in reshaping our understanding of apicomplexan evolutionary history (Videvall 2019). Despite their paramount significance, the genetic insights into these parasites remain limited. Most available data pertain to partial fragments of the mitochondrial cytochrome b (*cytb*) gene, commonly employed as a barcode for parasite identification. Remarkably, the MalAvi database (Bensch et al. 2009) houses over 4800 distinct lineages as of July 2023. However, the distinctiveness of these lineages, whether they present species or haplotypes, is frequently uncertain. Accurate species delimitation among avian malaria parasites holds pivotal implications for understanding ecological processes such as community assembly (Clark et al. 2018), as well as macroevolutionary dynamics, including host-switching versus cospeciation (Ricklefs et al. 2014). Galen et al. (2018) stated that an integrative coalescent approach amalgamating *cytb* barcode, morphology, ecological indicators (e.g., host specialization), and an array of genetic markers (21 nuclear genes) provides a highly accurate species delimitation. Contemporary descriptions of these parasites have expanded to include partial fragments of the mitochondrial cytochrome oxidase subunit 1 (COI), the nuclear DNA apical membrane antigen-1 (AMA1), and the Apicoplast gene (*clpc*) for molecular characterization (Valkiunas et al. 2017). Augmenting genetic data appears indispensable for accurate species demarcation and addressing broader phylogenetic inquiries, such as the timing of the haemosporidian radiation. However, amassing such data is fraught with challenges.

Two principal obstacles hinder the acquisition of additional genetic data. Firstly, the nature of the material under scrutiny—typically bird blood samples from wild individuals—poses complexities. These avian hosts may either harbor emerging infections, primarily observable in blood samples of *Plasmodium* species due to their asexual erythrocytic replication, or chronically infected cases where parasitemia remains exceedingly low (usually less than one parasite per 1000 erythrocytes) (Valkiunas 2005). This, coupled with the disparity in concentration between host and parasite DNA and the need to store samples in DNA-damaging lysis buffers, impedes successful DNA fragment amplification, particularly for larger fragments.

The second challenge arises from the prevalence of mixed infections. In natural settings, birds often harbor mixed infections comprising haemosporidian parasites of differing genera or even the same genus. Amplifying distinct gene fragments (e.g., nuclear and apicoplast genes) from such samples complicates the assignment to individual parasites due to the absence of comparative data.

Only a handful of studies have managed to amplify complete genomes (e.g., Bensch et al. 2016; Böhme et al. 2018) or entire mitochondrial genomes (e.g., Perkins 2008; Omori et al. 2008; Pacheco et al. 2018b) of avian haemosporidian parasites. Given the substantial resources required for amplifying and sequencing complete mitochondrial genomes, this endeavor will likely persist as a specialty endeavor.

This study introduces a highly sensitive nested PCR assay designed to simplify the amplification of entire mitochondrial genomes of avian haemosporidian parasites, even during the chronic infection stage of wild birds. The efficacy of this novel assay was rigorously assessed using known single-infected bird blood and tissue samples from previous investigations.

## Material and methods

### Samples

The samples used in this study comprise bird blood or tissue samples from previous studies that have detected single infections (Schmid et al. 2017; Musa et al. 2022; Magaña Vázquez et al. 2022). The blood samples (*n* = 134) belong to different Malagasy bird species collected in the Maromizaha rainforest in Eastern Madagascar, namely the Forest Fody (*Foudia madagascariensis*), Red Fody (*F. omissa*), Madagascar Bulbul (*Hypsipetes madagascariensis*), Ashy Cuckooshrike (*Coracina cinerea*), Paradise Flycatcher (*Terpsiphone mutata*), and different species of the Vangidae family (Table 1). Tissue samples of heart and liver belong to different Corvoidea species from southern Germany, the Eurasian Magpie (*Pica pica*, *n* = 3) and Carrion Crow (*Corvus corone*, *n* = 20) (Table 1). DNA was extracted from tissue samples using the Zymo Research extraction kit (Quick-gDNA™ MiniPrep; Zymo Research Europe GmbH, Freiburg, Germany) and from blood samples using the QIAamp DNA Blood Mini Kit (QIAGEN, Hilden, Germany) and then stored at −20 °C until further use. Concentration of DNA extracted from blood samples was between 0 and 50 ng/μL whereas tissue samples had concentrations of about 50 to 200 ng/μL.

**Table 1:** Potentially single-infected bird blood and tissue samples identified from previous studies. Bird family, species, and lineages of those samples are given with name and number in parentheses

Bird family	Bird species	Material	Lineage (*n*)
Campephagidae	*Coracina cinerea*	blood	lFOUOMI07 (3)
Monarchidae	*Terpsiphone mutata*	blood	lCINSOV02 (1), lTERMUT01 (1), pCOPALB03 (2)
Corvidae	*Corvus corone*	heart, liver	lCOCOR03 (3), lCOCOR09 (9), lCOCOR13 (5), lCOCOR17 (1), lCOCOR20 (1)
	*Pica pica*	heart, liver	pSGS1 (1), lPICPIC01 (1), lPICPIC02 (1)
Pycnonotidae	*Hypsipetes madagascariensis*	blood	hBUL2 (7), lBRALEP01 (1), lFOMAD01 (4), lFOUOMI02 (2), lHYPMA02 (3), lHYPMA03 (1), pBUL07 (9), pGRW09 (2), pHYPMA01 (2)
Ploceidae	*Foudia* spp.	blood	hFOUMAD02 (20), hRBQ11 (9), lFOMAD01 (1), lHYPMA02 (7), lNEWAM03 (1), lPHICAS01 (1), pCOLL4 (1), pCOLL7 (23), pFOUMAD03 (1), pGRW04 (4), pGRW09 (5), pWW3 (2)
Vangidae	*Cyanolanius madagascarinus*	blood	hCYAMAD01 (1)
	*Newtonia amphicroa*	blood	hNEWAM04 (3), lFOMAD01 (1), pNEWAM05 (1), pNEWAM06 (3), pNEWAM07 (3)
	*Newtonia brunneicauda*	blood	hNEWBR04 (1), hNEWBR05 (1), pNEWAM06 (1)
	*Calicalicus madagascariensis*	blood	lANLAT11 (1), lCALMAD03 (1), lHYPMA02 (1)
	*Pseudobias wardi*	blood	hPSEWAR01 (1), hPSEWAR03 (1)

### Design of nested PCR primers

**Fig. 1:**
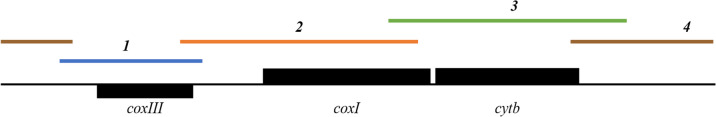
Schematic illustration of the linear mitochondrial genome of *Haemoproteus* sp. BO6 (KY200985). The three protein coding genes are shaded in black. Genes on the complementary strand are depicted below the line. Overlapping amplification products of the four nested PCRs are given with the associated number

Primers for the nested PCR approach were designed by aligning several published whole mitochondrial sequences in Geneious v. 2021.1.1 (https://www.geneious.com), which included *Plasmodium relictum* (NC_012426), *P. berghei* (NC_015303), *P.vinckei* (LR865437), *Haemoproteus* sp. BO6 (KY200985), *H. columbae* (KY653761), *Leucocytozoon caulleryi* (AB302215), *L. sabrazesi* (NC_009336), *L. fringillinarum* (KT162004), and *L. dubreuili* (KY653795). As the PCR approach is only suitable for single-infected samples, primer pairs were designed at homologous sites of the mitochondrial genome, shared by all haemosporidian genera. Four different overlapping fragments of about 1300–1900 bp serve as target sequences with overlapping zones of about 200 bp (Fig. 1).

### Nested PCR assay

PCR reactions of the first PCR were carried out in a total volume of 25 μL, containing 2.5 μL 10X ReproFast PCR Buffer with 20 mM MgSO4 (GENAXXON bioscience GmbH, Ulm, Germany), 1 μL of each primer (10 mM; Table 2), 0.5 μL of each dNTP (10 μmol), 0.125 μL ReproFast DNA polymerase (5 U/μL; GENAXXON bioscience GmbH, Ulm, Germany), 5 μL template DNA (10–100 ng/μL), and 14.875 μL nuclease-free water. The reaction mixture of the nested PCRs consisted of 5 μL 10X ReproFast PCR Buffer with 20 mM MgSO4 (GENAXXON bioscience GmbH, Ulm, Germany), 2 μL of each primer (10 mM; Table 2), 1 μL of each dNTP (10 μmol), 0.25 μL ReproFast DNA polymerase (5 U/μL; GENAXXON bioscience GmbH, Ulm, Germany), 2 μL amplification product of the initial PCR, and 37.75 μL nuclease-free water in a total volume of 50 μL. Cycling conditions of the different PCRs were performed as described in Table 2. Amplification products (5μL) of the nested PCRs were mixed with GelRedTM stain and then visualized on a 1.5% agarose gel after 20 min at 90 V.

**Table 2:** Nested PCR primers and cycling conditions used to amplify whole mitochondrial genome of haemosporidian parasites

PCR	Forward (F) and reverse (R) PCR primers	Modified after	Fragment size (bp)	Cycle conditions: temperature (°C)/time (s) for denaturation, annealing and extension steps
1 outer	F: AGACGTTCTTAACCCAGCTCACG	346F (Perkins 2008)	1.450	94/30, 58/30, 72/100
	R: CCTTTCCGGCTGTTTCCATCTC			
1 inner	F: ATTGGTAAGGTATAGCGTTTACTATCG		1.300	94/30, 57/30, 72/100
	R: GACGAGCGGTGTGTACAAGGC			
2 outer	F: CCAACAGAAAAATATTTTAAAGATG		2.050	94/30, 48/30, 72/120
	R: GAACATATCATATTCCAACCATTTA			
2 inner	F: ATAAAGAATATTATTTATAAGAACGG		1.900	94/30, 48/30, 72/120
	R: CCAAGGAAATGCATAGGTAA			
3 outer	F: TTTATATGTACATTTACTTTTGGWGG		2.020	94/30, 51/30, 72/120
	R: CTTGGAAGATTCGTAATTAGTGG			
3 inner	F: GCTTTACATGATACATATTATGTAATTGC	= 3180F (Perkins 2008)	1.900	94/30, 53/30, 72/120
	R: GAATATAGACGGTTTTCTGCG			
4 outer	F: TTAGCAAGACATGACAGGG		1.810	94/30, 51/30, 72/100
	R: GGAAAAGGAAAGGTTAACCGC			
4 inner	F: GGCAAGTTAAAGAAGTTCTGGTTT		1.750	94/30, 56/30, 72/100
	R: GGGAAGTGTGTTTCCATAGAAACCTTC	626R (Perkins 2008)		

All outer reactions included an initial denaturation period of 4 min at 94 °C and 25 cycles. Nested reactions included an initial denaturation period of 4 min at 94 °C and 35 cycles. All reactions included a final extension period of 7 min at 72 °C

**Fig. 2:**
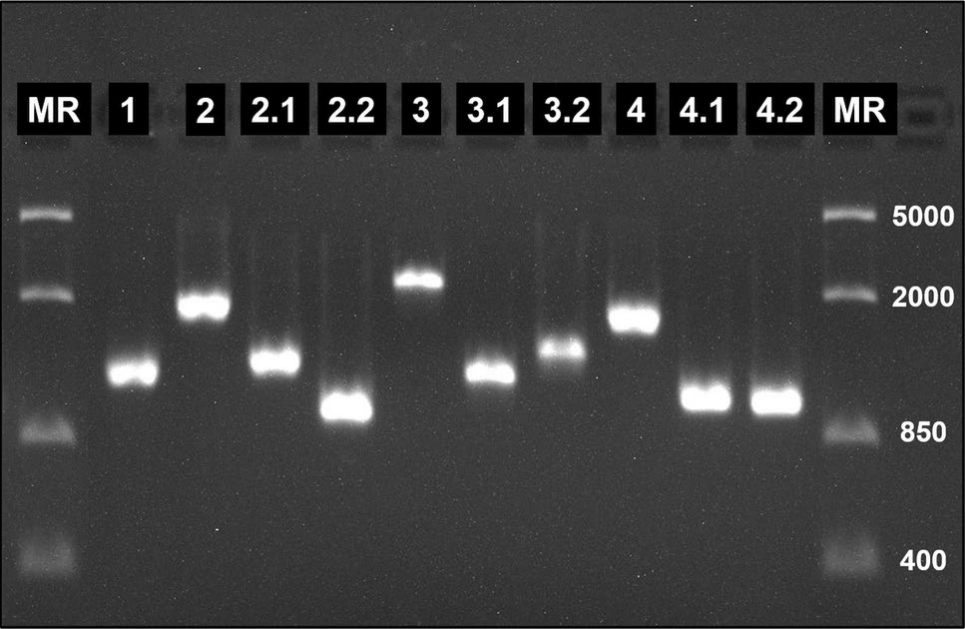
Agarose gel electrophoresis of nested PCR products of lFOMAD01 from *Hypsipetes madagascariensis* (Pycnonotidae) from Madagascar. Fragment numbers are given. Lane MR: molecular marker (FastRuler Middle Range DNA Ladder by Thermo Scientific™)

In most cases, successful amplification of the four fragments was possible. However, some problems occurred with DNA extracts from blood samples stored in lysis buffer. Large fragments 2, 3, and 4 were sometimes amplified in insufficient quantity and quality, affecting detection in the agarose gel and sequencing. For this reason, a PCR approach has been developed to amplify these fragments in two smaller, overlapping fragments (2.1/2.2, 3.1/3.2, and 4.1/4.2; Fig. 2). Reaction mixtures of the nested PCRs were the same as for the other PCRs. Primers and cycling conditions are given in Table 3.

**Table 3:** Nested PCR primers and cycling conditions used to amplify two smaller, overlapping fragments of large fragments no. 2, 3, and 4

PCR	Forward (F) and reverse (R) PCR primers	Modified after	Fragment size (bp)	Cycle conditions: temperature (°C)/time (s) for denaturation, annealing and extension steps
2.1	**F: ATAAAGAATATTATTTATAAGAACGG**		1.315	94/30, 48/30, 72/80
	R: GGATGTCCAAAGAACCAGAA			
2.2	F: ACTGGATGGACTTTATATCC		940	94/30, 48/30, 72/60
	**R: CCAAGGAAATGCATAGGTAA**			
3.1	**F: GCTTTACATGATACATATTATGTAATTGC**	= 3180F (Perkins 2008)	1.080	94/30, 53/30, 72/60
	R: GCATTATCTGGATGTGATAATGGT	= HaemR2 (Bensch et al. 2000)		
3.2	F: TTACCTTGGGGTCAAATGAG		1.190	94/30, 50/30, 72/80
	**R: GAATATAGACGGTTTTCTGCG**			
4.1	**F: GGCAAGTTAAAGAAGTTCTGGTTT**		1.070	94/30, 53/30, 72/60
	R: TTGAATGGAGCACTGGATTGG	5939R (Perkins 2008)		
4.2	F: ATCCTTAAATCTCGTAACCATGC	F2 (Pacheco et al. 2018b)	1.050	94/30, 56/30, 72/60
	**R: GGGAAGTGTGTTTCCATAGAAACCTTC**	626R (Perkins 2008)		

All outer reactions included an initial denaturation period of 4 min at 94 °C and 25 cycles. Nested reactions included an initial denaturation period of 4 min at 94 °C and 35 cycles. All reactions included a final extension period of 7 min at 72 °C

Amplification products were purified using the PCR Product Purification Kit (Roche, Mannheim, Germany). After sequencing (Microsynth AG, Balgach, Switzerland), the resulting sequence data were checked and edited using Geneious v. 2021.1.1 (https://www.geneious.com). The different fragments were mapped to a reference sequence (*Plasmodium relictum* (NC_012426) for *Plasmodium* lineages, *Haemoproteus* sp. BO6 (KY200985) for *Haemoproteus* lineages, or *Leucocytozoon caulleryi* (AB302215) for *Leucocytozoon* lineages) and the fragments were put together. If there were differences in the overlap regions or double nucleotide peaks were detected, then the sample was considered to contain a mixed infection. The resulting sequence was aligned to the originally identified *cytb* barcode of the sample to assure the similarity. If dissimilarities were recognized, the sequence was identified using the BLAST search of the MalAvi database (Bensch et al. 2009). Sequences were deposited in GenBank (OR327000-327004; OR347658-347675).

### Phylogenetic analysis

Nucleotide alignment was produced using MUSCLE alignment as implemented in Geneious. The alignment was constructed with a total of 53 mtDNA genome sequences, including all sequences obtained in this study and those available from GenBank (Benson et al. 2013). Sequences of the three protein coding genes were extracted and concatenated, keeping their order in the mtDNA genome (*cox3*, *cox1*, and *cytb*; 3341 bp). The phylogeny was generated by implementing the best fitting model (GTR+G+I) identified by MEGA v.10.2 (Kumar et al. 2018). The maximum likelihood method was carried out using 1000 replicates and MEGA v.10.2 was used to view and edit the resulting phylogram.

## Results

Complete mitochondrial genomes of 24 different avian haemosporidian lineages were successfully amplified (Table 4). For the other 19 lineages, it was not possible to amplify the entire genome because either double infections were detected or the original DNA isolate ran out to perform all the necessary PCRs.

**Table 4:** Avian haemosporidian lineages (name and Acc. No.) of which complete mitochondrial genome (Acc. No. comp Mt DNA) was successfully amplified. Bird host and site are given for each lineage

Genus	lineage	Acc. No.	bird host	Acc. No. comp Mt DNA	site
*Haemoproteus*	hBUL2	JN661910	*Hypsipetes madagascariensis*	OR327000	Madagascar
	hFOUMAD02	JN661941	*Foudia* spp.	OR326999	Madagascar
	hNEWAM04	MF442575	*Newtonia amphicroa*	OR327001	Madagascar
	hNEWBR04	MF442598	*Newtonia brunneicauda*	OR327002	Madagascar
	hNEWBR05	MF442601	*Newtonia brunneicauda*	OR327003	Madagascar
	hRBQ11	HQ386235	*Foudia* spp.	OR327004	Madagascar
*Leucocytozoon*	lCINSOV02	MF442615	*Terpsiphone mutata*	OR347658	Madagascar
	lCOCOR09	KJ128987	*Corvus corone*	OR347659	Germany
	lFOMAD01	JN032605	*Hypsipetes madagascariensis*	OR347660	Madagascar
	lHYPMA02	MF442609	*Foudia* spp.	OR347661	Madagascar
	lHYPMA03	MF442624	*Hypsipetes madagascariensis*	OR347662	Madagascar
	lPHICAS01	MF442616	*Foudia* spp.	OR347663	Madagascar
	lPICPIC01	MF189970	*Pica pica*	OR347664	Germany
	lPICPIC03*	-	*Pica pica*	OR347665	Germany
*Plasmodium*	pBUL07	JN661996	*Hypsipetes madagascariensis*	OR347666	Madagascar
	pCOLL4	KC867664	*Foudia* spp.	OR347667	Madagascar
	pCOLL7	JN661986	*Foudia* spp.	OR347668	Madagascar
	pCOPALB03	MF442560	*Terpsiphone mutata*	OR347669	Madagascar
	pFOUMAD03	JN661983	*Foudia* spp.	OR347670	Madagascar
	pGRW04	DQ839016	*Foudia* spp.	OR347671	Madagascar
	pHYPMA01	MF442542	*Hypsipetes madagascariensis*	OR347672	Madagascar
	pNEWAM05	MF442544	*Newtonia amphicroa*	OR347673	Madagascar
	pNEWAM07	MF442549	*Newtonia amphicroa*	OR347674	Madagascar
	pWW3	DQ847262	*Foudia* spp.	OR347675	Madagascar

*Newly detected lineage

**Fig. 3:**
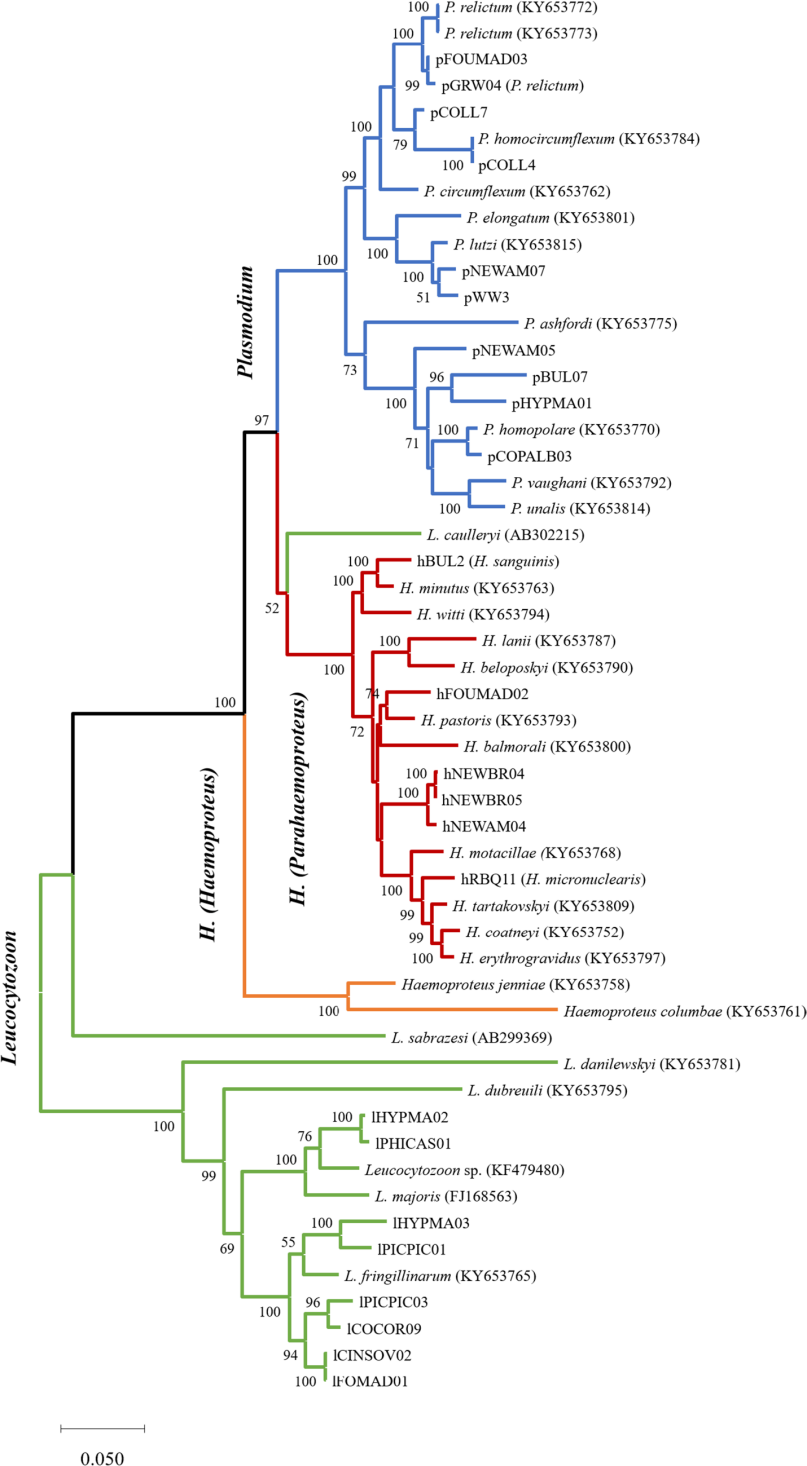
Phylogenetic relationship of haemosporidian parasites based on concatenated sequences of protein coding regions (*cox3*, *cox1*, and cytb). The analysis comprised 53 sequences (3341 bp) of morphospecies (Pacheco et al. 2018b) and newly amplified whole mitochondrial genomes. Values at the nodes are bootstrap values as a percentage obtained for 1000 replicates from a maximum likelihood tree (GTR+G+I). Branch colors indicate the haemosporidian genus. Species names are indicated in bold

The phylogenetic analysis revealed monophyly of all three haemosporidian genera (*Plasmodium*, *Haemoproteus*, and *Leucocytozoon*) and of the subgenera *Haemoproteus* and *Parahaemoproteus* (Fig. 3)*. Leucocytozoon* lineages lHYPMA02 and lPHICAS01 isolated from Malagasy birds group together with the morphospecies *Leucocytozoon majoris* while all other lineages group together with the morphospecies *L. fringillinarum*. *Haemoproteus* lineage hBUL2 is closely related with *H. minutus* (85 bp difference) while lineage hFOUMAD02 shows close relationship with *H. pastoris* (118 bp difference). *Haemoproteus* lineages isolated from *Newtonia* spp. (hNEWAM04, hNEWBR04, and hNEWBR05) form a separate, distinct clade. *Plasmodium* lineages show close relationships to various *Plasmodium* morphospecies. Lineages pGRW04 and pFOUMAD03 showed a difference of 18 bp and both form a closely related sister-clade to the other *P. relictum* sequences. The lineages pWW3 and pNEWAM07 form another sister-clade to *Plasmodium lutzi*. pNEWAM05, pBUL07, and pHYPMA01 are the only sequences showing no close relationship to previous described morphospecies.

## Discussion

In the present study, we have successfully developed a novel nested PCR assay designed to amplify entire mitochondrial genomes of avian haemosporidian parasites. This methodology was evaluated using distinct sets of samples possessing varying levels of DNA quality from wild birds. Encouragingly, complete mitochondrial genomes were effectively amplified from both frozen tissue samples and small blood samples preserved in lysis buffer.

The established nested PCR approach facilitates the amplification of complete mitochondrial genomes from avian hosts captured in the wild, even during the chronic infection phase (e.g., Pacheco et al. 2018a, b; Böhme et al. 2018). In particular, this method offers a simplified approach that can readily be incorporated into standard screening protocols. A crucial requirement for achieving successful amplification is the utilization of a single-infected sample, a factor that is inevitably discerned as part of routine screening processes. For identifying single infections, we recommend the multiplex PCR developed by Ciloglu et al. (2023), enabling the simultaneous detection of avian haemosporidian parasites across all three genera, including the subgenera *Haemoproteus* and *Parahaemoproteus*. Alternatively, the widely employed nested PCR assay by Hellgren et al. (2004) for specific amplification of *Plasmodium/Haemoproteus* and *Leucocytozoon cytb* barcodes, combined with the multiplex PCR by Pacheco et al. (2018a) for detecting mixed infections with *Plasmodium* and *Haemoproteus*, can be utilized. The multiplex PCR by Ciloglu et al. (2023) offers the advantage of streamlined execution and furnishes a robust indicator of multiple infections. Conversely, the combination of the methods by Hellgren et al. (2004) and Pacheco (2018a) yields not only identification of single infections but also a wealth of additional data, notably the parasite barcodes.

Through the new PCR approach, it emerged that many samples previously identified as single infections in prior studies (Musa et al. 2022; Magaña Vázquez et al. 2022; Schmid et al. 2017) in fact contained mixed infections. Notably, the tissue samples from Carrion Crows displayed a higher prevalence of multiple *Leucocytozoon* lineage infections (>65%) than initially anticipated (Schmid et al. 2017). Within these samples, successful amplification of the complete mitochondrial genome was only feasible for one lineage (lCOCOR09), as the majority (19 out of 20) demonstrated mixed infections. The capacity to identify parasites at the lineage level is restricted to fragment 3, covering the *cytb* barcode. When other fragments deviate from fragment 3 in overlapping regions, detection of mixed infections is possible, but precise identification of the additional parasite lineage is impeded due to data comparability limitations. Enhanced data availability in the future may enable identification of individual fragments and the resolution of mixed infections within genera.

Due to a lower amount of mixed infections, mitochondrial genome amplification was notably more successful using DNA extracts from tissue samples of Eurasian Magpies and blood samples from Malagasy birds stored in lysis buffer. Remarkably, over 55% of the lineages yielded successful amplification of their entire mitochondrial genomes. This achievement has produced a substantial corpus of new genetic data, significantly enriching the dataset for future phylogenetic inquiries. However, it is important to note that this method also requires a significant amount of DNA, and in five cases, we found that DNA quantity posed a significant limiting factor.

The reconstructed haemosporidian phylogeny closely mirrors the structure of the most recent hypothesis (Pacheco and Escalante 2023). *Leucocytozoon* species underpin the phylogenetic tree, with the exception of *Leucocytozoon (Akiba) caulleryi*, which forms a sister-clade to the subgenus *Parahaemoproteus*. This affinity may stem from shared vector usage, notably biting midges (Ceratopogonidae) (Pacheco and Escalante 2023). Within the *Leucocytozoon* cluster, the newly discovered lineages from Madagascar and Germany demonstrate no close ties to previously described morphospecies, suggesting the potential presence of undescribed or genetically unlinked morphospecies. Further investigations should address this intriguing observation.

In the *Haemoproteus* (*Parahaemoproteus*) clade, lineage hFOUMAD02 potentially represents an uncharacterized species. A distinct clade encompassing lineages hNEWBR04, hNEWBR05, and hNEWAM04 presents intriguing similarity, with hNEWAM04 differing by 31–33 base pairs, while hNEWBR04 and hNEWBR05 vary by just four base pairs out of a total of 3317. Morphological variations observed by Magaña Vázquez et al. (2022) in the gametocytes of these lineages suggest the likelihood of novel species. The postulation that hNEWAM04 may correspond to the previously defined *Haemoproteus vangii* (Savage et al. 2009) requires formal description in future studies.

Most *Plasmodium* lineages exhibit limited association with previously documented morphospecies, indicating potential novel species. Lineage pCOPALB03 appears closely linked to *P. homopolare*, a widespread New World parasite of Passeriformes (Walther et al. 2014). However, the sequences differ in 46 of 3317 base pairs, and distinctions in the meront morphology (Magaña Vázquez et al. 2022) suggest pCOPALB03 might constitute a separate species.

*Plasmodium relictum* stands out due to its ubiquitous distribution and extensive range of avian hosts and mosquito vectors. Within this species, five lineages (pSGS1, pGRW4, pGRW11, pLZFUS01, and pPHCOL01) were identified and partially characterized. These closely related lineages are indistinct morphologically and often cannot be differentiated using vector or blood stage morphology (Valkiunas et al. 2018). Unlike most other *Plasmodium* parasites, transmission of *P. relictum* pSGS1 takes place as far north as northern Norway (Marzal et al. 2011). In Europe, the findings of the lineage pGRW04 are restricted to tropical migratory birds after they return from winter quarters, suggesting the absence of active transmission on breeding grounds (Martínez-de la Puente et al. 2021). The reported differences in geographical distribution of the lineages pSGS1 and pGRW11 on the one hand and pGRW04 on the other are difficult to explain bearing in mind the enormously broad range of their susceptible avian hosts and globally distributed mosquito vector species, such as *Culex pipiens* and *Culex quinquefasciatus*. Looking at partial sequences of merozoite surface protein 1 (*msp1*) gene revealed differences in five alleles were revealed between the lineage pGRW04 and the lineages pSGS1 and pGRW11, suggesting the lack of gene flow between those parasites (Hellgren et al. 2015). Furthermore, preliminary observations indicate that several European bird species can resist pGRW04 strains, which were isolated from African migrating Great read warblers *Acrocephalus arundinaceus* (Dimitrov et al. 2015). This data indicates that the lineages pSGS1, pGRW4, pGRW11, pLZFUS01, and pPHCOL01 might belong to the same *P. relictum* morphotype, but some of them also might represent cryptic species of the *P. relictum* group. Phylogenetic analysis of whole mitochondrial genomes previously contained only sequences of pSGS1 (KY653773) and pGRW11 (KY653772). This study’s phylogenetic tree now includes data from pGRW04 and the quite similar pFOUMAD03 (21 bp difference of a total of 5997 bp). The branch containing sequences of pSGS1 and pGRW11 appears as sister-clade to the branch of pGRW04 and pFOUMAD03. Based on the concatenated sequence of protein coding genes (3317 bp), the lineages differ in 43–52 base pairs. Because of this clear separation of pGRW04 from pSGS1 in terms of genetic data and transmission areas, it is strongly suggested that these lineages be considered cryptic species.

In summary, our newly introduced PCR protocol enables the amplification of complete mitochondrial genomes from avian haemosporidian parasites. The assay provides a streamlined approach to obtaining extensive genetic data even from single-infected wild bird samples with mild parasitemia. This dataset proves pivotal for future phylogenetic analyses and species delimitation, as exemplified by our findings for pGRW04.

## Data Availability

All generated data (sequences) are published in GenBank (OR327000-327004; OR347658-347675).
